# Sociodemographic and clinicopathologic characteristics of patients treated with high dose rate prostate brachytherapy in Nigeria

**DOI:** 10.3332/ecancer.2024.1740

**Published:** 2024-08-14

**Authors:** Abbas A Abdus-Salam, Mutiu A Jimoh, Ayorinde M Folasire, Atara I Ntekim, Olabisi T Ojo, Chiamaka G Ehiedu, Sikiru A Yusuf, Augustine O Takure, Bidemi I Akinlade, Olusola K Idowu, Afolabi A Oladeji, Foluke O Sarimiye, Adeniyi A Adenipekun

**Affiliations:** 1 Department of Radiation Oncology, University of Ibadan and University College Hospital, Ibadan PMB 5116, Nigeria; 2 Department of Radiation Oncology, University College Hospital, Ibadan PMB 5116, Nigeria; 3 Department of Surgery, University of Ibadan and University College Hospital, Ibadan PMB 5116, Nigeria; 4 Department of Anaesthesia, University of Ibadan, Ibadan, Nigeria

**Keywords:** prostate cancer, PSA, routine screening, high dose rate brachytherapy

## Abstract

**Introduction:**

Prostate cancer is the most commonly diagnosed malignancy in adult males. High dose rate brachytherapy (HDRB) recently became available in the country for the management of localized prostate cancer in addition to other treatment modalities. HDRB offers a less invasive option to radical prostatectomy and also has a better side effects profile.

**Aim:**

To report the socio-demographic features of the patients treated with HDRB, the clinicopathologic pattern of their disease and possible predictors of these features.

**Methods:**

A retrospective study of patients with histopathologically confirmed prostate cancer, who had HDRB at the Department of Radiation Oncology, University College Hospital, Ibadan, Nigeria, between July 2020 and 2023 was done. Patients’ socio-demographic and clinicopathologic characteristics were extracted from their treatment records.

**Results:**

A total of 73 patients had HDRB within the period under review. The median age was 66 years (51–78 years). About 40% had prostate cancer diagnosed following routine prostate-specific antigen (PSA) screening. The median screening PSA was 20.5 ng/mL (5.83–75.35 ng/mL). About a quarter (24.7%) were asymptomatic at presentation while frequency (60.3%), nocturia (45.2%) and urgency (35.6%) were the most common symptoms. The median initial PSA was 26.00 ng/mL (5.10–124.50 ng/mL) and the median PSA before brachytherapy was 6.25 ng/mL (0.03–175.30 ng/mL), the majority (75.3%) had androgen deprivation therapy before brachytherapy. Forty-seven patients (64.4%) presented with TNM stages 1 and 2 while grade group 2 (24.7%) and high risk (76.7%) were the commonest grade group and risk group, respectively. There was a significant association between age group and TNM stage (*p* = 0.043), level of education (LOE) and TNM stage (*p* = 0.037) as well as PSA screening and grade group (*p *= 0.007).

**Conclusion:**

The majority of the patients who presented for prostate brachytherapy were elderly, had higher initial PSA, were in the high-risk group and had early-stage disease. About 25% of the patients were asymptomatic at presentation. Patients with tertiary LOE and elderly patients presented more with stage 1 and 2 diseases.

## Introduction

At 1.5 million new cases and 396,773 deaths worldwide in 2022, prostate cancer was the second most common cancer to be diagnosed and a leading cause of cancer-related death among males [1]. With an estimated 18,019 new cases and 11,443 deaths in Nigeria in 2022, prostate cancer was the most frequently diagnosed cancer and the primary cause of cancer-related death among males in Nigeria [2]. Nigeria accounted for 17.7% and 20.8% of the new prostate cancer cases and fatalities in Africa in 2022 [2,3].

Prostate cancer is considered a cancer of the elderly with the median age at presentation in Nigeria to be 66 years [4] and most of the patients presenting with adenocarcinoma of the prostate gland [5]. Ahmed *et al* [4] noted that 33% of 197 prostate cancer patients presented with organ-confined disease and 84.2% of the patients had high-risk disease.

There has been a significant increase in awareness of prostate cancer and willingness to screen among Nigerian men to about 51%–53% [6,7] when compared with what was obtainable in 2009 where 28.7% of men had good knowledge of prostate cancer and only 4.5% of them have been screened [8]. With awareness of routine serum prostate-specific antigen (PSA) screening now widespread, more patients now have an early diagnosis of prostate cancer with patients presenting more frequently with localized disease [9].

High-dose rate (HDR) interstitial brachytherapy can be used for localized prostate cancer management [10–13] and optimal treatment, requires adequate patient selection and risk stratification. The stage, grade and PSA are significant clinicopathologic features of prostate cancer, used in risk stratification. The National Comprehensive Cancer Network defines the following risk stratification: Low risk: T1-T2a, Gleason 2-6 and PSA <10 ng/mL, Intermediate risk: T2b-T2c, Gleason 7 or PSA 10–20 ng/mL and High risk: T3a, Gleason 8-10 or PSA >20 ng/mL [14].

High-dose rate brachytherapy (HDRB) can be used as monotherapy or in combination with EBRT and/or androgen deprivation therapy (ADT), based on risk stratification or as salvage therapy in advanced symptomatic cases [14–19].

The first HDRB equipment was installed in Nigeria in 2019 and this current study is the first of its kind in relation to prostate cancer and HDR treatment coming out of Nigeria. The HDR unit is a remote afterloading system supplied by BEBIG, Germany. The equipment comprises a SagiNova^®^ 25-channel HDR machine, a treatment planning system (SagiPlan^®^) and a customized ultrasound scanning system with rectal probe, templates, plastic and steel needles while the radionuclide that was used is Colbalt-60. Further details of the equipment and procedures used are described in Abdus-Salam *et al* [20].

### Aim

This study aims to report the socio-demographic, clinicopathologic and predictors of clinicopathologic characteristics of patients with prostate cancer who had prostate brachytherapy at the Department of Radiation Oncology, University College Hospital, Ibadan, Nigeria.

## Methods

The study is a descriptive retrospective study of patients with histopathologically confirmed prostate cancer who had high dose rate prostate brachytherapy at the Department of Radiation Oncology, University College Hospital, Ibadan, between July 2020 and 2023. Patients’ socio-demographic (age, tribe, marital status, level of education (LOE), religion and state of residence) and clinicopathologic characteristics (Routine PSA screening, PSA screening value, symptoms, initial PSA (PSA before commencement of any form of treatment), use of ADT and the type of ADT used, PSA value before brachytherapy, TNM staging (PSA and grade group were not used), Gleason score, grade group and risk group) were extracted from the patients’ treatment records. The collected data were analyzed using SPSS v. 22. Results were presented in prose and tables. The student’s *t*-test was used for quantitative variables and the chi-squared test or Fisher’s exact test was used to test for association between possible predictors and clinicopathologic characteristics. Kolmogorov-Smirnov test was used to test for normality for age, initial PSA and Gleason score while Spearman’s Rho test was used to test for correlation between age, initial PSA and Gleason score. Linear regression analysis was used to test for association between age, initial PSA and Gleason score. The level of significance was set at 0.05. Ethical approval was obtained from, the joint ethical review committee of the University of Ibadan and University College Hospital, Ibadan.

## Results

A total of 73 patients had HDR prostate brachytherapy within the period under review. The socio-demographic variables are as detailed in Table 1. The median age of the patients was 66 years (range: 51–78 years) and forty-five patients (61.6%) were over 64 years. Over half of the patients were from the Yoruba tribe (54.8%) and resided in the southwestern part of Nigeria (58.9%). The majority of the patients were married (91.8%), had a tertiary LOE (79.5%) and practiced Christianity (86.3%). 

Twenty-nine patients (39.7%) had prostate cancer diagnosed following routine PSA screening, among whom, 37.9% had symptoms on direct questioning and almost half (48.3%) had screening PSA value greater than 20 ng/mL. The median screening PSA was 20.5 ng/mL and ranged from 5.83 to 75.35 ng/mL. Almost a quarter (24.7%) were asymptomatic at presentation while frequency (60.3%), nocturia (45.2%), urgency (35.6%) and weak stream (26.0%) were the most common symptoms among symptomatic patients. Forty-four patients (60.3%) had initial PSA (PSA before any treatment) >20 ng/mL. Fifty-five patients (75.3%) had ADT with androgen receptor blockers (ARBs) being the most common (56.4%). About half of the patients (54.8%) had a PSA value of less than 10 ng/mL before the brachytherapy procedure. The median initial PSA at presentation was 26.00 ng/mL and ranged from 5.10 to 124.50 ng/mL and the median PSA just before brachytherapy was 6.25 ng/mL and ranged from 0.03 to 175.30 ng/mL. Forty-seven patients (64.4%) presented with TNM stages 1 and 2, and the most common histopathologic grade group and risk group were grade group 2 (24.7%) and high-risk group (76.7%), respectively. The clinicopathologic characteristics were are detailed in Tables 2 and 3.

**Figure 1.**
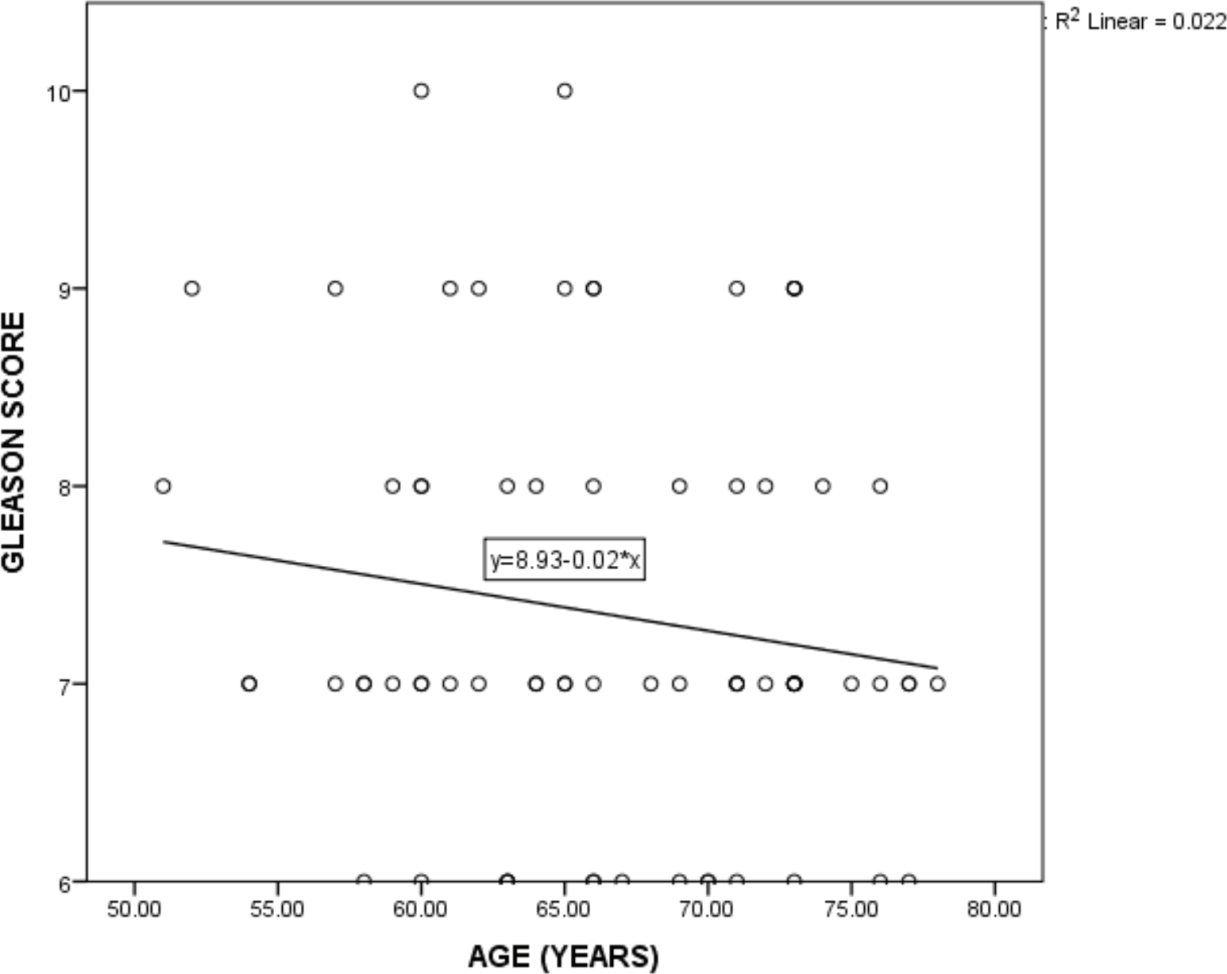
Regression plot of age and Gleason score.

**Figure 2.**
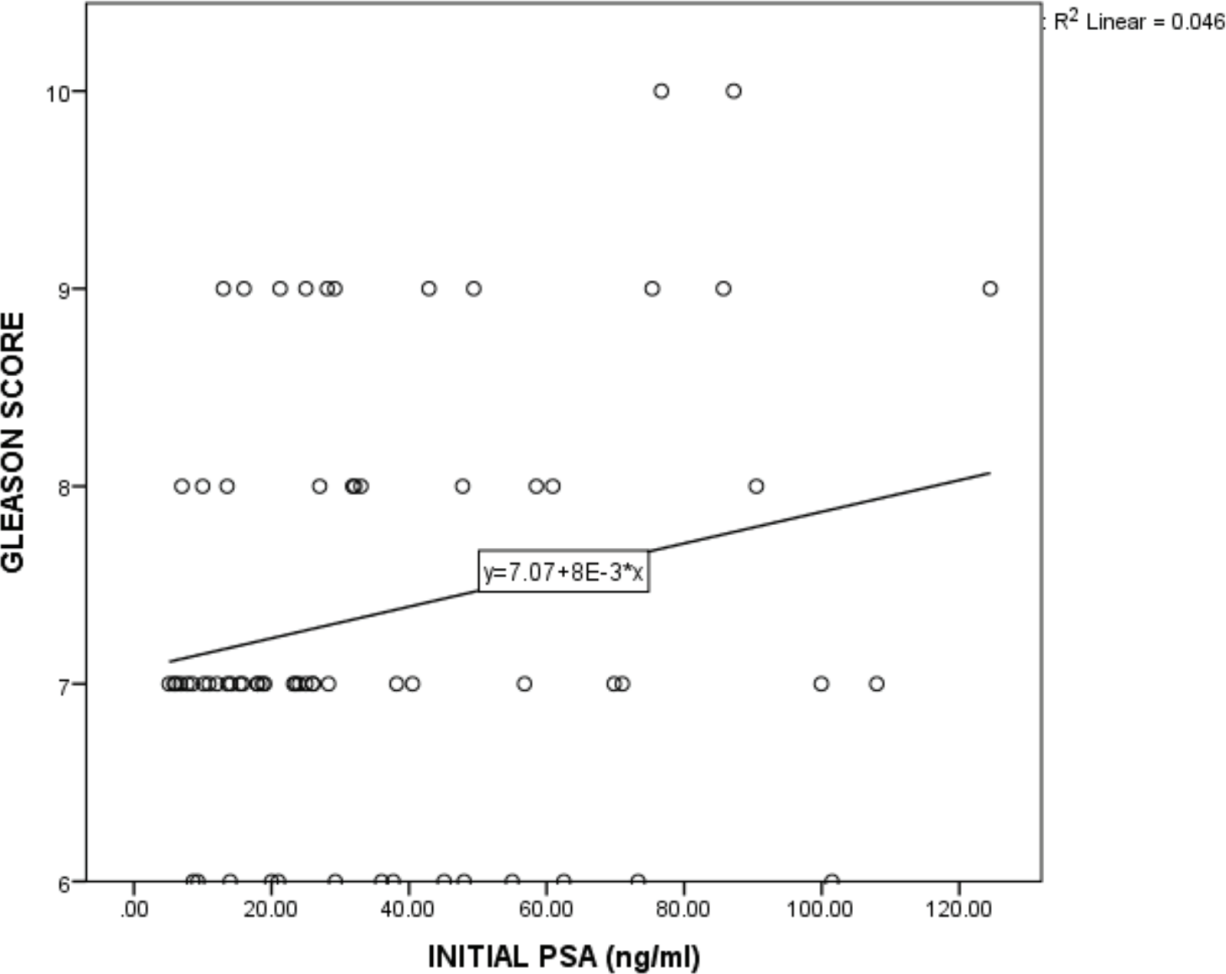
Regression plot of initial PSA and Gleason score.

A higher proportion of patients older than 64 years (73.3%) presented with TNM stages 1 and 2 compared with those between ages 51 and 64 years (50%) and this association was statistically significant (*p* = 0.043). No statistically significant relationship was found between age group and PSA screening, PSA screening value, initial PSA, grade group and risk group (Table 4). There was a slight negative correlation between age and Gleason score though not significant (r_s_ = −0.116, *p* = 0.168, R2 = 0.022) (Figure 1). There was no statistically significant relationship between residential zone and PSA screening, PSA screening value, initial PSA, TNM stage, grade group and risk group (Table 5). A higher percentage of patients with tertiary LOE (61.5%) presented with TNM stage 1 and 2 diseases compared with those with primary (40.0%) or secondary LOE (50.0%) and this association was statistically significant (*p* = 0.037). No statistically significant relationship was found between the LOE and PSA screening, PSA screening value, initial PSA, grade group and risk group (Table 6). A higher proportion of patients who were Christians (59.1%) had PSA screening values greater than 20 ng/mL compared to the Muslims who had a higher proportion (83.3%) with PSA screening values between 10 and 20 ng/mL and this association was statistically significant (*p* = 0.016). No statistically significant relationship was found between religion and PSA screening, initial PSA, TNM stage, grade group and risk group (Table 7). More of the patients who had PSA screening presented with grade group 1 and 2 diseases (25.0% and 46.4%, respectively) compared with those who did not and had more of grade group 3, 4 and 5 diseases (23.3%, 20.9% and 25.6%, respectively). This association was statistically significant (*p* = 0.007). Test of association between PSA screening and initial PSA, TNM stage and risk group were not statistically significant (Table 8). There was no statistically significant relationship between initial PSA and TNM stage, grade group and risk group (Table 9). There was a non-significant slightly positive correlation between the initial PSA and Gleason score (r_s_ = 0.150, *p* = 0.109, R2 = 0.046) (Figure 2).

## Discussion

The median age of patients in this study was 66 years (range: 51–78 years) with 61.6% being over 64 years. This finding is similar to those of other studies carried out among patients with prostate cancer presenting for prostate brachytherapy which showed median ages ranging from 64 to 71 years [21–25]. This is not unexpected as the incidence of prostate cancer increases with increasing age [10].

Over half of the patients were of Yoruba origin (54.8%) and resided in the Southwestern part of Nigeria (58.9%). This is expected as the facility is located in the Southwestern part of Nigeria where Yoruba is the major ethnic group. This finding is in tandem with those of a study done in Lagos, Nigeria, among patients with prostate cancer [26], though not presenting for prostate brachytherapy. Patients also came from other parts of Nigeria for the procedure and this could be because high dose rate prostate brachytherapy is only available in Nigeria at the Department of Radiation Oncology, University College Hospital, Ibadan, as at the time of this report. 

The majority of the patients (91.8%) were married and this is similar to findings from other studies done in Nigeria [26, 27]. This could be explained by the sociocultural practices in Nigeria as shown by the demographic health survey in Nigeria which demonstrated that the percentage of married men increases with increasing age [28] and is not necessarily a hazard posed by marriage. Being married has however been shown to have a lesser risk of late presentation, under-treatment and death [29]. 

This study showed that the majority (79.5%) of the patients had a tertiary LOE and this was in tandem with the study done by Adewumi *et al* [26]. This could be attributed to an increased level of awareness which may be due to the literacy status, hence presenting at an early stage which is an indication for prostate brachytherapy. The majority (86.3%) of the patients were Christians and this was also demonstrated by other studies carried out among prostate cancer patients in Lagos and Oyo States [26, 30]. An unexplained significant positive correlation between the practice of Christianity and prostate cancer incidence and a significant negative correlation between the practice of Islam and prostate cancer incidence has been demonstrated [31].

Twenty-nine patients (39.7%) were diagnosed following PSA screening in this study. This could be due to increasing awareness and acceptance of PSA screening by Nigerian men [32]. This study also revealed the median screening PSA value to be 20.5 ng/mL (range: 5.83–78.35 ng/mL) with almost half (48.3%) of the patients having screening PSA value greater than 20 ng/mL. This finding is similar to another study done in Lagos, which showed a PSA screening value greater than 20 ng/mL in 55.8% of patients with prostate cancer diagnosis following screening [33]. This could be because blacks are more prone to have elevated PSA levels and more aggressive diseases [34–37]. Among those who were diagnosed following routine PSA screening, 37.9% had symptoms on direct questioning. This could be due to a lack of awareness of symptoms of prostate cancer or attribution of symptoms to other prostate conditions such as benign prostatic hyperplasia. 

Almost a quarter (24.7%) were asymptomatic at presentation and this emphasizes the need for routine PSA screening. Among those with symptoms, frequency (60.3%), nocturia (45.2%), urgency (35.6%), weak stream (26.0%), feeling of incomplete voiding (24.7%) and hesitancy (20.5%) were the most common symptoms. This finding is in tandem with findings from other studies done in Osun, Lagos, Rivers and Cameroon [26, 38–40]. However, in contrast to findings from these studies, symptoms of distant metastasis were absent in our study as distant metastasis (especially poly-metastasis) is a contraindication for brachytherapy procedures.

Our study showed that the median initial PSA was 26.0 ng/mL (range: 5.10–124.5 ng/mL) with 44 patients (60.3%) having PSA value greater than 20 ng/mL, which classifies them as high risk. This finding is in tandem with results from other studies done in Jos, Ghana and Cameroon which revealed the proportion of patients with initial PSA greater than 20 ng/mL to be 74.0%, 60.7% and 51.4%, respectively [40–42]. The median PSA before brachytherapy was in our study was 6.25 ng/mL (range: 0.03–175.3 ng/mL) with 15 patients (20.5%) having a PSA value greater than 20 ng/mL. This finding is similar to results from other studies which revealed median PSA before brachytherapy to be 7.7 ng/mL and 11.9 ng/mL in the UK and Japan, respectively [43, 44], and the proportion of patients with PSA greater than 20 ng/mL before brachytherapy to be 20.7%, 29.6%, 22.2% and 17.6% in UK, Japan, Ghana and Brazil, respectively [24, 25, 43, 44]. This is not unexpected as some patients, especially those with locally advanced disease commenced ADT before brachytherapy. In our study, 75.3% of the patients had ADT before brachytherapy, with 56.4% having ARBs. This finding is in tandem with studies done in the UK and Australia where 69.2% and 88%, respectively, had neoadjuvant ADT [21, 43].

Our study revealed that 47 patients (64.4%) presented with stage 1 or 2 disease. Studies done in the UK, Australia and Japan revealed similar findings as 72.0%, 70.5% and 66.0% of patients who had brachytherapy had T1/T2 disease, respectively [21, 43, 45]. This finding differs from the results of studies done in Lagos and Cameroon which revealed that 10.4% and 9.5% of patients with prostate cancer, presented with stage 1 or 2 disease, respectively [26, 46]. This difference is because brachytherapy as a treatment option is indicated for localized disease, hence more patients with stage 1 or 2 disease in our study.

Forty-six patients (63.0%) in our study had Gleason scores of 6 or 7 (Grade group 1–3) and this is similar to results from other studies done in the UK, USA and Japan which revealed that 66.2%, 77.0% and 81.7% of patients for brachytherapy had Gleason score 6 or 7, respectively [23, 43, 44]. This finding also explains the early-stage disease found in this cohort of patients as prostate cancer with a Gleason score less than or equal to 7 is less aggressive compared to those greater than 7. Also, this study showed that 76.7% of patients were high-risk. This finding is contrary to the results from 2 studies done in the UK and a study done in Taiwan which revealed that 54.0%, 52.3% and 50.0% of the patients presenting for prostate brachytherapy were high risk, respectively [43, 47, 48]. This difference could be because blacks tend to have higher PSA levels [34–36] and a high percentage (60.3%) of patients from our study had initial PSA greater than 20 ng/mL which classified them as high risk.

There was a statistically significant association between age group and stage of presentation (*p* = 0.043) in our study as a higher proportion of patients over 64 years of age (73.3%) presented with stage 1 or 2 disease compared to those within 51–64 years of age (50.0%). There has been an increase in the incidence of prostate cancer among men 55 years and below, and the patients within this age group are less likely to have organ-confined disease [49]. This supports the school of thought that young African men with prostate cancer usually have an aggressive disease and this could be due to genetic mutation [50], hence the need for more genetic studies in our setting.

In this study, the association between LOE and stage of presentation was statistically significant (*p* = 0.037) as a higher percentage of patients with tertiary LOE presented with stage 1 or 2 disease compared with those with primary LOE (40.0%) and secondary LOE (50.0%). This could be because men with tertiary LOE tend to be screened [51], have better knowledge of prostate cancer [52] and opt for curative procedures [53].

The association between PSA screening and grade group was also statistically significant (*p* = 0.007) as a higher proportion of patients who had PSA screening presented with grade 1 (25.0%) or grade 2 (46.4%) compared to those who did not have PSA screening who presented with grade 1 (18.6%) or 2 (11.6%) disease. This finding is similar to the results of a study that revealed that a higher proportion of patients who presented for PSA screening had prostate cancer with a Gleason score less than or equal to 6 compared to those who did not (*p* < 0.001) [54]. This could be due to the detection of cancer in its early stage following screening as it has been shown that an increase in the grade of prostate cancer can occur especially within the first 2 years of diagnosis [55].

## Limitations

Due to the retrospective nature of this study, some data such as PSA values and grade group were missing and could not be reported. The low sample size is also a limitation of this study. Further prospective studies that will explore the sociodemographic and disease characteristics of prostate cancer patients presenting for brachytherapy in Nigeria as well as the effect of these factors on survival are recommended. In addition, further studies on the outcome of the treatment including biochemical controls are currently being worked on. We also hope to compare our outcomes with those of operative procedures including prostatectomies.

## Conclusion

This study showed that over half of the patients who presented for prostate brachytherapy were over 64 years old. Most of the patients presented with higher PSA values and were in the high-risk group. The most common grade group were grade groups 1 and 2 and the most common stages were stages 1 and 2 while 24.7% of the patients were asymptomatic. Patients over 64 years and patients with a tertiary LOE presented more with early-stage disease (stages 1 and 2). 

This observation calls for more PSA screening and prostate cancer awareness programs especially among young male adults, as this will enable diagnosis of asymptomatic disease and early detection of prostate cancer symptoms which translates to organ-confined disease that will benefit from prostate brachytherapy.

## Conflicts of interest

The authors declare that they have no conflict of interest.

## Funding

None.

## Consent for publication

All authors agreed on the publication of this manuscript.

## Ethical consideration

All procedures performed in studies involving human participants were in accordance with the ethical standards of the Institutional and/or National Research Committee and with the 1964 Helsinki Declaration and its later amendments or comparable ethical standards. The study was approved by the joint ethical Committee of the University of Ibadan and University College Hospital Ibadan.

## Availability of data and materials

The datasets generated during and/or analyzed during the current study are available from the corresponding author upon reasonable request.

## Author contributions

All authors contributed to the study conception and design. All authors took part in the management of all the patients. Data collection was done by Olabisi T Ojo, Akanni S Yusuf and Chiamaka G Ehiedu under the supervision of Abdus-Salam A A and Jimoh M A. Data analysis was done by Chiamaka G Ehiedu and reviewed by Abdus-Salam A A. The first draft of the manuscript was written by Olabisi T Ojo and Chiamaka G Ehiedu and all authors revised and commented on several versions of the manuscript. The final manuscript represents the aggregate of revisions and adjustments by all the authors. It was approved by all the authors.

## Figures and Tables

**Table 1.** Sociodemographic characteristics.

Variables (*N*= 73)	Frequency	Percent
Age range (years)		
51–64	28	38.4%
>64	45	61.6%
Tribe		
Yoruba	40	54.8%
Igbo	25	34.2%
Bini	3	4.1%
Esan	2	2.7%
Urhobo	1	1.4%
Ibibio	1	1.4%
Isoko	1	1.4%
Residential zone		
South West	43	58.9%
South East	17	23.3%
Abuja	7	9.6%
South South	5	6.8%
North Central	1	1.4%
Marital status		
Married	67	91.8%
Widower	3	4.1%
Divorced	1	1.4%
Single	1	1.4%
Seperated	1	1.4%
LOE		
Primary	5	6.8%
Secondary	2	2.7%
Tertiary	58	79.5%
Not stated	8	11.0%
Religion		
Christian	63	86.3%
Islam	10	13.7%

**Table 2.** Clinicopathologic characteristics.

Variables	Frequency	Percent
PSA screening (*N* = 73)		
Yes	29	39.7%
No	44	60.3%
Symptoms present on direct questioning among those elevated PSA on screening (*N* = 29)		
Yes	11	37.9%
No	18	62.1%
Screening PSA range (*N* = 29)		
<10	5	17.2%
10–20	9	31.0%
>20	14	48.3%
Missing	1	3.4%
Symptoms (*N* = 73)		
Asymptomatic	18	24.7%
Frequency	44	60.3%
Nocturia	33	45.2%
Urgency	26	35.6%
Weak stream	19	26.0%
Incomplete voiding	18	24.7%
Hesitancy	15	20.5%
Dysuria	14	19.2%
Straining	13	17.8%
Terminal dribbling	11	15.1%
Low back pain	11	15.1%
Retention	7	9.6%
Intermittency	5	6.8%
Hematuria	4	5.5%

**Table 3.** Clinicopathologic characteristics.

Variables	Frequency	Percent
Initial PSA (ng/mL) (*N* = 73)		
<10	9	12.3%
10–20	18	25.7%
> 20	44	60.3%
Missing	2	2.7%
ADT (*N* = 73)		
Yes	55	75.3%
No	18	24.7%
ADT type (*N* = 55)		
ARB	31	56.4%
GNRH agonist	11	20.0%
ARB + GNRH agonist	8	14.5%
Orchidectomy	1	1.8%
ORCHIDECTOMY + ARB	2	3.6%
ORCHIDECTOMY + GNRH agonist	1	1.8%
Not specified	1	1.8%
PSA before brachytherapy (ng/mL) (*N* = 73)		
<10	40	54.8%
10–20	15	20.5%
> 20	15	20.5%
Missing	3	4.1%
TNM staging (*N* = 73)		
Stage 1 and 2	47	64.4%
Stage 3	17	23.3%
Stage 4A	6	8.2%
Stage 4B	3	4.1%
Grade group (*N* = 73)		
1	15	20.5%
2	18	24.7%
3	13	17.8%
4	12	16.4%
5	13	17.8%
Missing	2	2.7%
Risk group (*N* = 73)		
Low/intermediate risk	15	20.5%
High risk	56	76.7%
Missing	2	2.7%

**Table 4.** Age and clinicopathologic characteristics.

Variable	Age range			*p*-value
	51–64	> 64	Total	
PSA screening				0.952*
Yes	11(39.3%)	18(40.0%)	29(39.7%)	
No	17(60.7%)	27(60.0%)	44(60.3%)	
PSA screening range (ng/mL)				0.872**
<10	1(10.0%)	4(22.2%)	5(17.9%)	
10–20	4(40.0%)	5(27.8%)	9(32.1%)	
>20	5(50.0%)	9(50.0%)	14(50.0%)	
Initial PSA (ng/mL)				0.480*
< 10	3(11.1%)	6(13.6%)	9(12.7%)	
10–20	9(33.3%)	9(20.5%)	18(25.4%)	
> 20	15(55.6%)	29(65.9%)	44(62.0%)	
Stage				0.043**^
Stage 1 and 2	14(50.0%)	33(73.3%)	47(64.4%)	
Stage 3	7(25.0%)	10(22.2%)	17(23.3%)	
Stage 4A	5(17.9%)	1(2.2%)	6(8.2%)	
Stage 4B	2(7.1%)	1(2.2%)	3(4.1%)	
Grade				0.844*
1	5(17.9%)	10(23.3%)	15(21.1%)	
2	8(28.6%)	10(23.3%)	18(25.4%)	
3	4(14.3%)	9(20.9%)	13(18.3%)	
4	6(21.4%)	6(14.0%)	12(16.9%)	
5	5(17.9%)	8(18.6%)	13(18.3%)	
Risk group				0.519*
High risk	21(75.0%)	35(81.4%)	56(78.9%)	
Low/intermediate risk	7(25.0%)	8(18.6%)	15(21.1%)	

* Chi square

** Fishers Exact

^ Statistically significant

**Table 5.** Residence and clinicopathologic characteristics.

Variable	Residence			*p*-value
	South	North/FCT	Total	
PSA screening				0.604**
Yes	26(40.0%)	3(37.5%)	29(39.7%)	
No	39(60.0%)	5(62.5%)	44(60.3%)	
PSA screening range (ng/mL)				1.000**
<10	5(20.0%)	0(0.0%)	5(17.9%)	
10–20	8(32.0%)	1(33.3%)	9(32.1%)	
>20	12(48.0%)	2(66.7%)	14(50.0%)	
Initial PSA (ng/mL)				0.752**
< 10	9(14.3%)	0(0.0%)	9(12.7%)	
10–20	16(25.4%)	2(25.0%)	18(25.4%)	
> 20	38(60.3%)	6(75.0%)	44(62.0%)	
Stage				0.505**
Stage 1 and 2	42(64.6%)	5(62.5%)	47(64.4%)	
Stage 3	15(23.1%)	2(25.0%)	17(23.3%)	
Stage 4A	6(9.2%)	0(0.0%)	6(8.2%)	
Stage 4B	2(3.1%)	1(12.5%)	3(4.1%)	
Grade				0.595**
1	14(22.2%)	1(12.5%)	15(21.1%)	
2	17(27.0%)	1(12.5%)	18(25.4%)	
3	12(19.0%)	1(12.5%)	13(18.3%)	
4	10(15.9%)	2(25.0%)	12(16.9%)	
5	10(15.9%)	3(37.5%)	13(18.3%)	
Risk group				1.000**
High risk	49(77.8%)	7(87.5%)	56(78.9%)	
Low/intermediate risk	14(22.2%)	1(12.5%)	15(21.1%)	

** Fishers Exact

**Table 6.** LOE and clinicopathologic characteristics.

Variable	LOE				*p*-value
	1^0^	2^0^	3^0^	Total	
PSA screening					0.701**
Yes	2(40.0%)	0(0.0%)	25(43.1%)	27(41.5%)	
No	3(60.0%)	2(100.0%)	33(56.9%)	38(58.5%)	
PSA screening range (ng/mL)					1.000**
<10	0(0.0%)	0(0.0%)	5(20.0%)	5(19.2%)	
10–20	0(0.0%)	0(0.0%)	9(36.0%)	9(34.6%)	
>20	1(100.0%)	0(0.0%)	11(44.0%)	12(46.2%)	
Initial PSA (ng/mL)					1.000**
<10	0(0.0%)	0(0.0%)	9(15.8%)	9(14.3%)	
10–20	1(25.0%)	0(0.0%)	14(24.6%)	15(23.8%)	
> 20	3(75.0%)	2(100.0%)	34(59.6%)	39(61.9%)	
Stage					0.037**^
Stage 1 and 2	2(40.0%)	1(50.0%)	37(63.8%)	40(61.5%)	
Stage 3	0(0.0%)	1(50.0%)	15(25.9	16(24.6%)	
Stage 4A	3(60.0%)	0(0.0%)	3(5.2%)	6(9.2%)	
Stage 4B	0(0.0%)	0(0.0%)	3(5.2%)	3(4.6%)	
Grade					0.364**
1	1(25.0%)	0(0.0%)	12(21.1%)	13(20.6%)	
2	0(0.0%)	1(50.0%)	15(26.3%)	16(25.4%)	
3	2(50.0%)	1(50.0%)	8(14.0%)	11(17.5%)	
4	1(25.0%)	0(0.0%)	10(17.5%)	11(17.5%)	
5	0(0.0%)	0(0.0%)	12(21.1%)	12(19.0%)	
Risk group					0.730**
High risk	4(100.0%)	2(100.0%)	44(77.2%)	50(79.4%)	
Low/intermediate risk	0(0.0%)	0(0.0%)	13(22.8%)	13(20.6%)	

** Fishers Exact

^ Statistically significant

**Table 7.** Religion and clinicopathologic characteristics.

Variable	Religion			*p*-value
	Christian	Islam	Total	
PSA screening				0.144**
Yes	23(36.5%)	6(60.0%)	29(39.7%)	
No	40(63.5%)	4(40.0%)	44(60.3%)	
PSA screening range (ng/mL)				0.016**^
<10	5(22.7%)	0(0.0%)	5(17.9%)	
10–20	4(18.2%)	5(83.3%)	9(32.%)1	
>20	13(59.1%)	1(16.7%)	14(50.0%)	
Initial PSA (ng/mL)				0.153**
<10	8(13.1%)	1(10.0%)	9(12.7%)	
10–20	13(21.3%)	5(50.0%)	18(25.4%)	
>20	40(65.6%)	4(40.0%)	44(62.0%)	
Stage				0.574**
Stage 1 and 2	41(65.1%)	6(60.0%)	47(64.4%)	
Stage 3	13(20.6%)	4(40.0%)	17(23.3%)	
Stage 4A	6(9.5%)	0(0.0%)	6(8.2%)	
Stage 4B	3(4.8%)	0(0.0%)	3(4.1%)	
Grade				0.739**
1	14(22.6%)	1(11.1%)	15(21.1%)	
2	14(22.6%)	4(44.4%)	18(25.4%)	
3	11(17.7%)	2(22.2%)	13(18.3%)	
4	11(17.7%)	1(11.1%)	12(16.9%)	
5	12(19.4%)	1(11.1%)	13(18.3%)	
Risk group				0.087**
High risk	51(82.3%)	5(55.6%)	56(78.9%)	
Low/intermediate risk	11(17.7%)	4(44.4%)	15(21.1%)	

** Fishers Exact

^ Statistically significant

**Table 8.** PSA screening and clinicopathologic characteristics.

Variable	PSA screening			*p*-value
	Yes	No	Total	
Initial PSA (ng/mL)				0.530*
< 10	5(17.9%)	4(9.3%)	9(12.7%)	
10–20	6(21.4%)	12(27.9%)	18(25.4%)	
> 20	17(60.7%)	27(62.8%)	44(62.0%)	
Stage				0.911**
Stage 1 and 2	19(65.5%)	28(63.6%)	47(64.4%)	
Stage 3	6(20.7%)	11(25.0%)	17(23.3%)	
Stage 4A	3(10.3%)	3(6.8%)	6(8.2%)	
Stage 4B	1(3.4%)	2(4.5%)	3(4.1%)	
Grade				0.007*^
1	7(25.0%)	8(18.6%)	15(21.1%)	
2	13(46.4%)	5(11.6%)	18(25.4%)	
3	3(10.7%)	10(23.3%)	13(18.3%)	
4	3(10.7%)	9(20.9%)	12(16.9%)	
5	2(7.1%)	11(25.6%)	13(18.3%)	
Risk group				0.268*
High risk	21(72.4%)	35(83.3%)	56(78.9%)	
Low/intermediate risk	8(27.6%)	7(16.7%)	15(21.1%)	

* Chi squared

** Fishers Exact

^ Statistically significant

**Table 9.** Initial PSA and clinicopathologic characteristics.

Variable	Initial PSA				*p*-value
	< 10	10–20	>20	Total	
Stage					0.140**
Stage 1 and 2	4(44.4%)	15(83.3%)	27(61.4%)	46(64.8%)	
Stage 3	4(44.4%)	2(11.1%)	11(25.0%)	17(23.9%)	
Stage 4A	0(0.0%)	0(0.0%)	5(11.4%)	5(7.0%)	
Stage 4B	1(11.1%)	1(5.6%)	1(2.3%)	3(4.2%)	
Grade					
1	2(22.2%)	2(11.8%)	10(23.3%)	14(20.3%)	0.361**
2	3(33.3%)	7(41.2%)	8(18.6%)	18(26.1%)	
3	3(33.3%)	4(23.5%)	6(14.0%)	13(18.8%)	
4	1(11.1%)	2(11.8%)	8(18.6%)	11(15.9%)	
5	0(0.0%)	2(11.8%)	11(25.6%)	13(18.8%)	

** Fishers Exact
